# Influence of Printing Parameters and Nozzle Diameter on the Effective Microarchitecture and Compressive Modulus of Gyroid PCL Scaffolds

**DOI:** 10.3390/jfb17060289

**Published:** 2026-06-09

**Authors:** José González, Mario E. Flores, Luis Medina Uzcátegui, Gabriela Martínez

**Affiliations:** 1 Escuela de Ingeniería Civil Mecánica, Facultad de Ciencias de la Ingeniería, Universidad Austral de Chile, Valdivia 5090000, Chile; jose.gonzalez05@alumnos.uach.cl; 2 Instituto de Ciencias Químicas, Facultad de Ciencias, Universidad Austral de Chile, Valdivia 5090000, Chile; mario.flores@uach.cl; 3 Instituto de Ingeniería Mecánica, Facultad de Ciencias de la Ingeniería, Universidad Austral de Chile, Valdivia 5090000, Chile; luis.medina@uach.cl

**Keywords:** gyroid scaffolds, polycaprolactone (PCL), fused deposition modeling, porosity, compressive elastic modulus, process–structure–property relationship

## Abstract

Three-dimensional scaffolds based on triply periodic minimal surfaces (TPMSs) have attracted growing interest in bone tissue engineering because of their high interconnectivity and ability to combine high porosity with mechanical integrity. However, in fused deposition modeling (FDM), printed architecture may systematically deviate from the nominal design, thereby affecting structural fidelity and mechanical performance. This study investigated the influence of FDM processing parameters and nozzle diameter on the effective microarchitecture and compressive elastic modulus of polycaprolactone (PCL) gyroid scaffolds. First, a Taguchi L18 design was used to evaluate the effects of extrusion temperature, printing speed, and flow rate on pore size for two nozzle diameters (0.4 and 0.3 mm). In a second experimental stage, prismatic specimens fabricated at three nominal porosity levels were compression-tested to determine the elastic modulus (E), and measured porosity (ϕ) was quantified by densimetric measurements. A systematic mismatch was observed between the nominal design and the printed scaffold architecture, with both pore size and measured porosity consistently lower than their intended values. The dominant process parameter associated with pore-size variability was nozzle-specific: extrusion temperature contributed most for the 0.4 mm nozzle, whereas printing speed contributed most for the 0.3 mm nozzle. In compression, E decreased with increasing measured porosity, and statistical analysis showed that the E–ϕ relationship was nozzle-dependent. Overall, these findings support a process–structure–property interpretation based on the effective printed microarchitecture rather than on nominal design parameters alone. The experimental stiffness ranges obtained here also provide an exploratory mechanical contextualization relative to reported trabecular bone domains, without implying site-specific scaffold selection.

## 1. Introduction

Tissue engineering seeks to restore or regenerate damaged tissues through the combined use of cells, biomaterials, and biochemical cues [1,2]. Within this field, three-dimensional scaffolds play a central role by providing structural support and a suitable environment for cell adhesion, proliferation, and differentiation. For clinical application, such scaffolds must be both biocompatible and biodegradable so that they can be gradually replaced by newly formed tissue [3,4].

Additive manufacturing has enabled the controlled fabrication of complex porous scaffolds with architectures that would be difficult to obtain using conventional methods. Among the available techniques, fused deposition modeling (FDM) is particularly attractive because of its low cost, accessibility, and compatibility with thermoplastic biomaterials [5,6,7]. Polycaprolactone (PCL), in turn, is widely used in bone tissue engineering because of its biocompatibility, relatively low melting temperature, and slow degradation rate, which allows it to provide mechanical support over extended periods. These characteristics make PCL especially suitable for scaffold applications requiring both manufacturability and structural stability [8,9,10].

Beyond the base material, the biological and mechanical performance of a scaffold is strongly influenced by its internal architecture. Parameters such as porosity, pore size, and pore interconnectivity govern key processes including cell infiltration, vascularization, and nutrient transport, while also influencing the overall stiffness of the structure [4,11,12,13]. In general, relatively high porosity levels, often above 50%, are regarded as beneficial for tissue ingrowth and mass transport, although excessive porosity may compromise mechanical support [11,13]. In bone tissue engineering, pore sizes ranging from approximately 200 to 1000 µm are commonly reported, with intermediate values often associated with enhanced vascularization and tissue ingrowth [4,12].

Scaffolds based on triply periodic minimal surfaces (TPMSs) have received growing attention because they can combine high porosity and interconnectivity with more uniform stress distributions [14,15,16,17,18]. Among these architectures, the gyroid is particularly promising because it forms a continuous, non-self-intersecting network that facilitates both nutrient transport and mechanical load distribution. Recent studies have shown that TPMS-based scaffolds can achieve porosity levels of approximately 50% to 90% while maintaining mechanical integrity and supporting vascularization through their highly interconnected pathways [15,16,17,18,19].

Despite the growing interest in TPMS-based scaffolds, the printed architecture of FDM-fabricated PCL scaffolds remains highly sensitive to processing conditions. Extrusion temperature, printing speed, and flow rate influence filament deposition and pore formation, which can cause the fabricated architecture to deviate systematically from the nominal CAD design [8,20,21,22]. As a result, the final scaffold may exhibit pore size and porosity values that differ substantially from those intended at the design stage [21,22]. Although some of these factors have been studied individually, a more integrated understanding of how processing parameters and nozzle diameter affect the effective printed microarchitecture and its relationship with compressive behavior in gyroid PCL scaffolds remains limited [6,7,8,10,20,23]. This limitation is especially relevant in bone tissue engineering, where scaffold performance depends strongly on the architecture actually obtained after fabrication.

The present work investigates how FDM processing parameters and nozzle diameter influence pore size and measured porosity in gyroid PCL scaffolds, and how the resulting printed microarchitecture relates to compressive stiffness. To address this, the study adopts a process–structure–property framework based on measured porosity rather than on nominal design parameters alone, thereby enabling a more realistic interpretation of scaffold performance. The findings are further discussed in terms of their exploratory mechanical relevance relative to stiffness domains reported for trabecular bone.

This study is limited to PCL scaffolds without chemical modifications or surface treatments to isolate the effects of the base polymer and the FDM process on the resulting microarchitecture and initial compressive response.

## 2. Methodology

### 2.1. Geometric Design of Scaffolds

**Figure 1:**
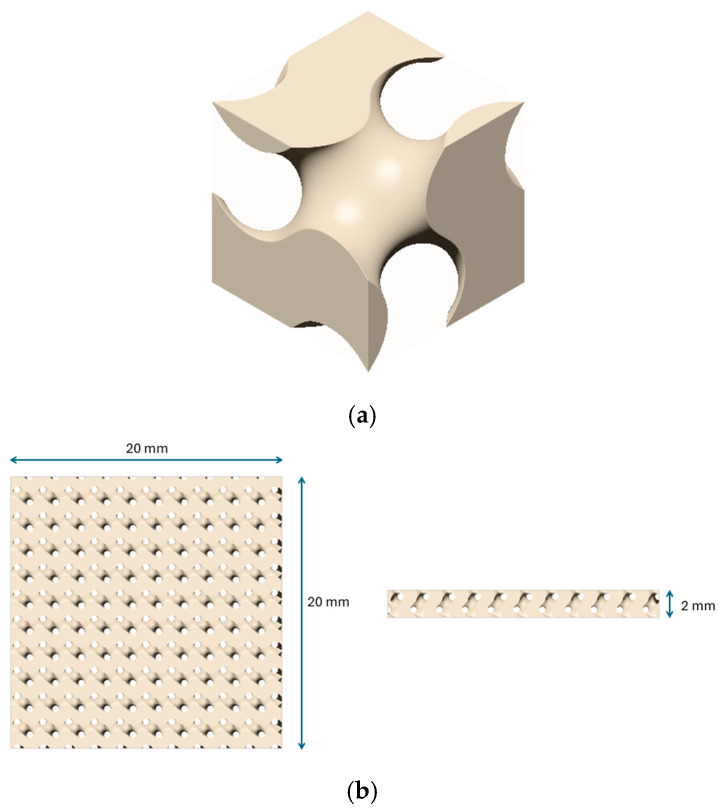

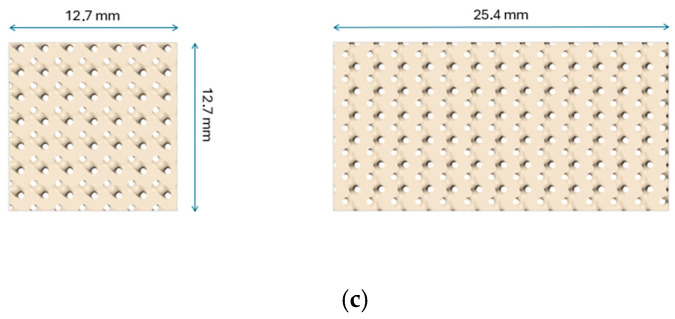
Geometry and dimensions of the specimens used in this study: (**a**) representative gyroid TPMS unit cell generated in Fusion 360 [24]; (**b**) plate scaffold for pore-size evaluation (DoE1); (**c**) prismatic specimen for compression testing (DoE2).

The scaffolds were designed using a gyroid architecture in Autodesk Fusion 360 (version 2025; Autodesk, Inc., San Francisco, CA, USA) [24]. A parametric TPMS template available within the platform was employed to generate periodic unit cells based on predefined geometric parameters. The representative gyroid TPMS unit cell used in this study is shown in Figure 1a.

Because the geometric resolution achievable in the printed structure depends on nozzle diameter and the resulting filament width during FDM fabrication, two-unit cell sizes were selected to preserve the gyroid pattern and avoid geometric discontinuities or local pore closure. Specifically, a unit cell size of 1.2 mm was used for the 0.4 mm nozzle, whereas a unit cell size of 0.9 mm was used for the 0.3 mm nozzle. These values are also consistent with ranges reported in the literature as suitable for bone tissue engineering applications [4,12].

Two types of specimens were defined for the experimental campaign. For the first experimental design (DoE1, pore-size analysis), plate-like scaffolds with dimensions of 20 × 20 × 2 mm were designed (Figure 1b) to evaluate pore size and its variability as a function of printing parameters while maintaining constant porosity. For the second experimental design (DoE2, mechanical characterization), prismatic specimens with dimensions of 12.7 × 12.7 × 25.4 mm were designed (Figure 1c) according to ASTM D695-15 recommendations for compression testing [25]. For DoE2, different nominal porosity levels were considered to assess their relationship with measured porosity and compressive stiffness. All models preserved the gyroid topology and were exported in STL format for subsequent FDM fabrication.

### 2.2. Fabrication by Fused Deposition Modeling (FDM)

The scaffolds were fabricated by fused deposition modeling (FDM) using a Creality Ender 3 printer (Shenzhen Creality 3D Technology Co., Ltd., Shenzhen, China) [26]. A natural polycaprolactone (PCL) filament with a diameter of 1.75 mm, supplied by Filaments.ca (Kitchener, ON, Canada), batch 1802021, was used for all specimens.

Three main process parameters were evaluated—extrusion temperature (T), printing speed (V), and flow rate (F)—because of their known influence on PCL deposition and scaffold geometry in FDM-fabricated structures [8,20,21,22]. For the 0.4 mm nozzle, the ranges considered were 80–120 °C for extrusion temperature, 5–20 mm/s for printing speed, and 100–110% for flow rate [8,21,22,27]. For the 0.3 mm nozzle, narrower ranges were adopted (90–110 °C, 5–10 mm/s, and 95–105%, respectively), since the smaller nozzle diameter makes deposition stability more sensitive to thermal and flow variations.

Table 1 summarizes the experimental levels of the FDM process parameters evaluated for each nozzle diameter: extrusion temperature (T), printing speed (V), and material flow rate (F). All other printing settings were kept constant throughout the experiments to isolate the effects of the selected factors. Slicing was performed in Ultimaker Cura 5.10.0 [28], and the corresponding G-code was generated for fabrication. Flow rate is reported as the Cura material-flow multiplier relative to the nominal extrusion calculated by the slicer.

A layer height of 0.20 mm was used with the 0.4 mm nozzle, whereas a layer height of 0.15 mm was used with the 0.3 mm nozzle. The line width was set equal to the nozzle diameter in each case. The heated bed, cooling fan, and retraction were disabled for all experiments. Infill density was set to 100%, so that porosity was determined exclusively by the TPMS geometry defined in the CAD model rather than by a Cura-generated infill pattern. Travel speed was set equal to the printing speed. The fixed slicing parameters used for both nozzle diameters are summarized in Table 2.

In all printing conditions, wall lines, top layers, and bottom layers were disabled. Therefore, no additional external/internal walls, roof, or floor were introduced during slicing, and the CAD-defined gyroid pore network was not intentionally modified by the slicer. The infill pattern was not used to generate the scaffold architecture; instead, infill density was set to 100% to fully fabricate the TPMS geometry previously defined in Fusion 360 [24]. No CAD-based additive manufacturing compensation, horizontal expansion, hole horizontal expansion, or G-code correction was intentionally applied. Thus, the reported deviations between nominal and measured pore size correspond to the uncompensated FDM fabrication of CAD-defined gyroid PCL scaffolds under the stated slicing conditions.

### 2.3. Experimental Design for DoE1 (Pore-Size Analysis)

To quantify the influence of printing parameters on the pore geometry of gyroid PCL scaffolds, a Taguchi design based on an L18 orthogonal array was applied independently to each nozzle diameter (0.4 and 0.3 mm). Table 3 summarizes the 18 experimental combinations evaluated for each nozzle, with extrusion temperature (T), printing speed (V), and flow rate (F) considered as control factors.

For each experimental condition, three plate-like specimens (20 × 20 × 2 mm) were fabricated. Pore size was determined from images acquired with a Leica S6D stereomicroscope (Leica Microsystems GmbH, Wetzlar, Germany) [29] by measuring the distance between adjacent filaments. Each specimen was imaged together with a millimeter scale, and image calibration was performed in ImageJ (version 1.54; National Institutes of Health, Bethesda, MD, USA) [30]. Three pore-size measurements were taken in internal regions of each specimen to avoid edge effects.

The three measurements obtained from each specimen were averaged to yield a single representative pore-size value per specimen, denoted ${\bar{P}}_{ij}$, where *i* identifies the experimental condition i=1,…,18 and j identifies the replicate within that condition (j=1,2,3). Thus, although nine raw measurements were collected for each condition, the statistical unit used in the subsequent analysis was the specimen-level average. Based on the three ${\bar{P}}_{ij}\;$values obtained for each condition, the mean pore size ${\bar{P}}_{i}\;$and the inter-specimen standard deviation $s_{i}$ were calculated, the latter being used as an indicator of geometric repeatability.

Because the nominal pore sizes were 0.8 mm for the 0.4 mm nozzle and 0.6 mm for the 0.3 mm nozzle, the relative error with respect to the target value for each condition was defined as follows:

(1) $$e_{i}=\;|\frac{{\bar{P}}_{i}-P^{*}}{P^{*}}|\;\times \;100$$

where $P^{*}\;$denotes the nominal pore size corresponding to each nozzle diameter.

The effects of T, V, and F on pore size were evaluated by analysis of variance (ANOVA), using the specimen-level averages ${\bar{P}}_{ij}\;$as experimental units (54 observations per nozzle). Given the available sample size per condition, the analysis was restricted to the main effects of the factors, without including interaction terms. The relative contribution of each factor to the observed variability was estimated as the ratio between the factor sum of squares and the total sum of squares. When a factor was found to be significant, differences between levels were further explored by post hoc comparisons at a significance level of α=0.05. Descriptive plots were also used to examine trends across factor levels.

To select representative printing conditions for DoE2, a multi-objective analysis was performed at the experimental-condition level by simultaneously minimizing the relative pore-size error $e_{i}$ and the inter-specimen standard deviation $s_{i}$. Candidate conditions were identified from the Pareto front. Among the non-dominated configurations, the primary ranking criterion was minimum relative error, whereas inter-specimen standard deviation was used as a secondary criterion to favor geometrically repeatable conditions. Manufacturability of the prismatic specimens was then used as a practical screening criterion for DoE2.

### 2.4. DoE2—Compressive Mechanical Properties

In the second experimental stage, the compressive response of FDM-fabricated gyroid PCL scaffolds was evaluated. For each nozzle diameter, one printing condition was selected from DoE1 by prioritizing combinations with low relative error with respect to the target pore size and low geometric variability. Accordingly, for each nozzle, a single combination of extrusion temperature (T), printing speed (V), and flow rate (F) was kept constant throughout DoE2. When the condition initially selected from the Pareto front did not allow stable fabrication of prismatic specimens, an alternative condition with comparable geometric performance in DoE1 and improved manufacturability was adopted.

Under these conditions, scaffolds were designed with three CAD-defined nominal porosity levels $\left(\phi_{nom}=40\%,\;50\%,\;\mathrm{and}\;60\%\right)$ to evaluate the influence of scaffold porosity on the compressive elastic modulus $\left(E\right)$. These nominal porosity values were controlled at the CAD stage by modifying the solid phase thickness of the gyroid architecture while keeping the unit cell size constant for each nozzle diameter (Section 2.1). After fabrication, the actual porosity of each specimen was experimentally determined by the densimetric method described below. The specimens were designed as prisms measuring 12.7 × 12.7 × 25.4 mm, in accordance with ASTM D695-15 recommendations for compression testing [25]. For each nozzle diameter–nominal porosity combination, three replicates were fabricated $\left(n=3\right)$.

Compression tests were performed under quasi-static conditions using an Instron 4469 universal testing machine equipped with a 50 kN load cell [31] at a constant crosshead speed of 2 mm/min, while recording the complete engineering stress–strain curve for each specimen. The compressive elastic modulus $\left(E\right)$ was calculated according to ASTM D695-15 as the slope of the initial linear region of the engineering stress–strain curve, obtained by linear fitting over the 0–2% strain interval for all specimens [25].

In addition, the measured porosity (ϕ) of each specimen was determined by a densimetric method:

(2) $$\phi =\left(1-\frac{\rho_{app}}{\rho_{PCL}}\right)\times 100$$

where the apparent density ($\rho_{app}$) was calculated as the ratio between the measured mass of each specimen and its external volume [32], and the theoretical density of PCL ($\rho_{PCL}$) was taken as 1.145 g/${cm}^{3}$ [33]:

(3) $$\rho_{app}=\frac{m}{V}$$

Subsequently, the relationship between measured porosity (ϕ) and compressive elastic modulus (E) was evaluated using a general linear model with interaction, in which ϕ was treated as a continuous variable and nozzle diameter ($d_{b}$) as a categorical variable:

(4) $$E=\beta_{0}+\beta_{1}\phi +\beta_{2}d_{b}+\beta_{3}(\phi \times d_{b})+\varepsilon$$

where $d_{b}=0$ for the 0.3 mm nozzle and $d_{b}=1$ for the 0.4 mm nozzle.

Additionally, a two-way ANOVA was performed on E, with nominal porosity level (40%, 50%, and 60%) and nozzle diameter treated as categorical factors, including the interaction term in the model. The linear model was used to describe the relationship between effective printed microarchitecture and mechanical response, whereas the ANOVA was used to test for differences across the nominal design levels defined at the CAD stage. In all statistical analyses, a significance level of α=0.05 was adopted.

## 3. Results

### 3.1. Pore-Size Analysis (DoE1)

#### 3.1.1. 0.4 mm Nozzle

**Figure 2:**
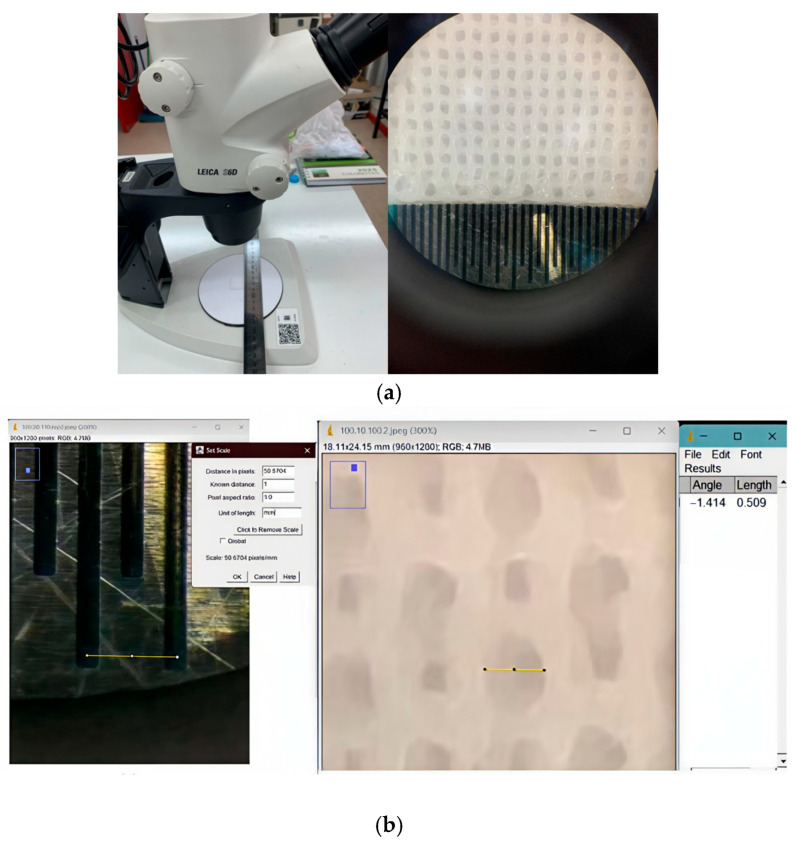
Microscopic image acquisition and pore-size measurement procedure: (**a**) specimen positioning and image capture with scale reference; (**b**) scale calibration and example of pore-size measurement for condition M4_10, specimen 2.

Pore-size measurements were performed as described in Section 2.3. Figure 2 illustrates the microscopic image acquisition, scale calibration, and pore-size measurement procedure used for the analyzed specimens.

Table 4 summarizes the results obtained for the 18 Taguchi L18 combinations evaluated with the 0.4 mm nozzle (M4_1–M4_18). For each condition, three specimens were fabricated, and three pore-size measurements were taken in internal regions of each specimen, yielding nine raw measurements per condition. As described in Section 2.3, these measurements were averaged at the specimen level before statistical analysis. The individual measurements are provided in Tables S1 and S2 of the Supplementary Material.

For all evaluated conditions, the mean pore size per condition, ${\bar{P}}_{i}$, remained below the nominal design value of 0.8 mm, ranging from 0.481 to 0.709 mm (Table 4). The relative error with respect to the target value ranged from 11% for condition M4_18 to 40% for M4_6. Condition M4_18 (120 °C, 20 mm/s, 110%) showed the best dimensional agreement, whereas M4_6 (80 °C, 20 mm/s, 100%) exhibited the largest deviation from the target pore size.

**Figure 3:**
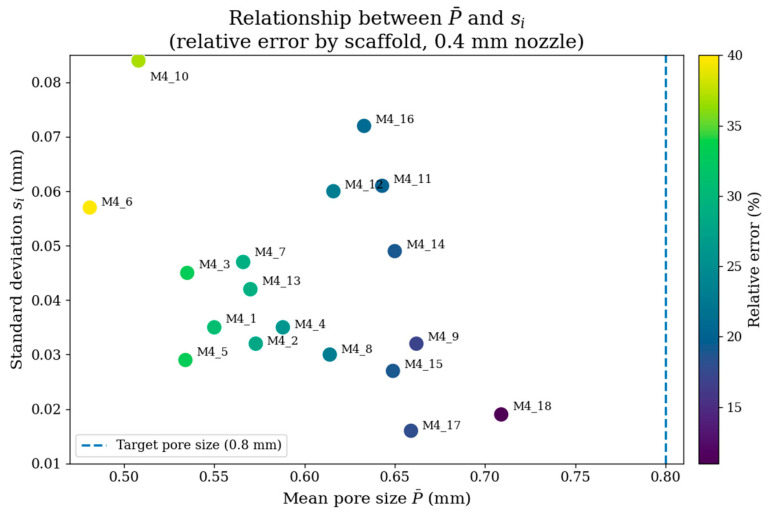
Relationship between mean pore size per condition and inter-specimen standard deviation for the 18 conditions printed with the 0.4 mm nozzle. Point color represents the relative error with respect to the target pore size.

Regarding geometric repeatability, the inter-specimen standard deviation, $s_{i}$, ranged from 0.016 mm (M4_17) to 0.084 mm (M4_10). Approximately 78% of the conditions showed standard deviations below 0.06 mm, indicating acceptable dimensional stability for most of the evaluated parameter combinations. Figure 3 shows the distribution of the 18 conditions as a function of mean pore size and inter-specimen standard deviation, with relative error represented by color.

ANOVA performed on the specimen-level average pore-size values (${\bar{P}}_{ij}$) showed that extrusion temperature was the dominant and highly significant factor affecting pore size (F=19.9, $p=5.439\times 10^{-7}$), whereas printing speed was also significant, although to a lesser extent (F=4.34, $p=1.84\times 10^{-2}$). In contrast, flow rate did not show a significant effect within the evaluated range (F=0.25, p=0.7825) (Table 5). In terms of relative contribution to the observed variability, temperature accounted for 41.5% of the total sum of squares, followed by printing speed with 9.1%, whereas flow rate contributed only 0.5%.

Finally, the multi-objective analysis identified three non-dominated conditions on the Pareto front: M4_18, M4_9, and M4_17. Among them, M4_18 showed the lowest relative error together with low geometric variability and was therefore selected as the reference condition for fabricating prismatic compression specimens with the 0.4 mm nozzle.

#### 3.1.2. 0.3 mm Nozzle

Table 6 summarizes the results obtained for the 18 Taguchi L18 combinations evaluated with the 0.3 mm nozzle (M3_1–M3_18). For each condition, three plate-like specimens were fabricated, and three pore-size measurements were taken in internal regions of each specimen, yielding nine raw measurements per condition. The individual measurements are reported in Tables S3 and S4 of the Supplementary Material.

As shown in Table 6, the mean pore size for every condition remained below the nominal design value of 0.6 mm, indicating a consistent mismatch between the CAD-defined geometry and the printed scaffold architecture. Mean values (${\bar{P}}_{i}$) ranged from 0.308 to 0.489 mm. The smallest relative error was obtained for M3_11 (100 °C, 10 mm/s, 100%), whereas M3_3 (90 °C, 7.5 mm/s, 95%) showed the largest deviation from the target pore size, with relative errors of 19% and 49%, respectively.

**Figure 4:**
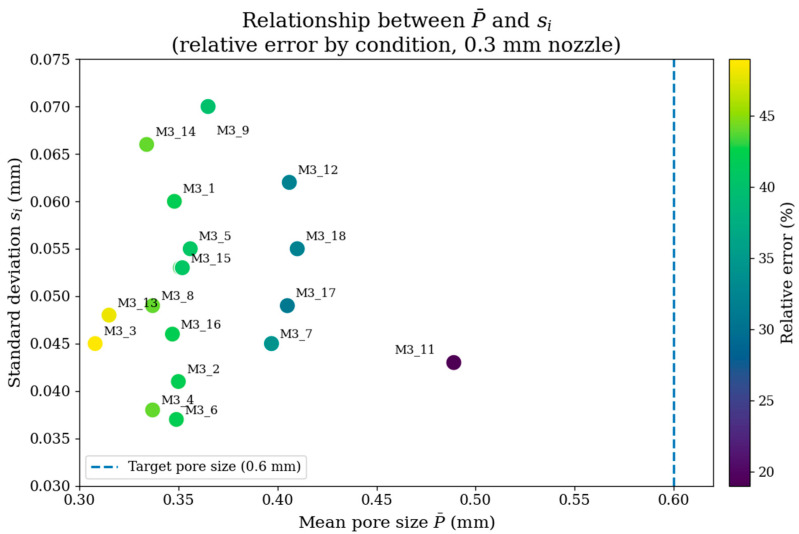
Relationship between mean pore size per condition and inter-specimen standard deviation for the 18 conditions printed with the 0.3 mm nozzle. Point color represents the relative error with respect to the target pore size.

Regarding geometric repeatability, the inter-specimen standard deviation, $s_{i}$, ranged from 0.016 mm (M3_16) to 0.064 mm (M3_12). Approximately 88% of the evaluated combinations showed standard deviations below 0.06 mm, indicating acceptable dimensional stability across most of the tested conditions. Figure 4 shows the distribution of the 18 combinations as a function of mean pore size and inter-specimen standard deviation, with relative error represented by color.

The ANOVA results are presented in Table 7. Among the factors examined, printing speed was the dominant factor affecting pore size and was highly significant in the 0.3 mm nozzle dataset (p<0.001). Temperature and flow rate were also statistically significant (p<0.05), although their contributions were substantially smaller. In terms of variance contribution, printing speed accounted for 31.6% of the total variation, whereas temperature and flow rate each contributed 8.2%. In contrast to the 0.4 mm nozzle, for which pore size was governed primarily by extrusion temperature, the 0.3 mm nozzle showed greater sensitivity to printing speed.

Based on the multi-objective analysis, three non-dominated conditions were identified on the Pareto front: M3_11, M3_17, and M3_18. Of these, M3_11 offered the most favorable trade-off between dimensional accuracy and geometric stability and was therefore selected for the fabrication of prismatic specimens in DoE2.

### 3.2. Compressive Response and Measured Porosity (DoE2)

#### 3.2.1. The 0.4 mm Nozzle

Based on the multi-objective analysis of DoE1, condition M4_18 (120 °C, 20 mm/s, 110% flow rate) was initially selected as the reference configuration for compression testing. However, during the fabrication of prismatic specimens (12.7 × 12.7 × 25.4 mm), recurrent defects were observed, including local collapse, geometric distortion, and loss of structural stability. These issues prevented the production of specimens compliant with ASTM D695-15, and this condition was therefore excluded from the mechanical testing campaign.

Reducing the extrusion temperature to 100 °C while maintaining printing speed and flow rate constant (20 mm/s and 110%, respectively), corresponding to the second non-dominated condition identified in the DoE1 Pareto front (M4_9), resulted in stable prismatic specimens free of visible macroscopic defects and compliant with ASTM D695-15 requirements. This parameter combination was therefore selected as the final condition for DoE2 with the 0.4 mm nozzle.

Under these conditions, nine specimens were fabricated across three nominal porosity levels ($\phi_{\mathrm{nom}}=40\%,\;50\%,\;60\%$; n=3 per level). The measured porosity (ϕ), determined by a densimetric method, was consistently lower than the nominal values. Specifically, measured porosity was approximately 28% for $\phi_{\mathrm{nom}}=40\%$, 37–38% for $\phi_{\mathrm{nom}}=50\%$, and 46–47% for $\phi_{\mathrm{nom}}=60\%$ (Table 8).

**Figure 5:**
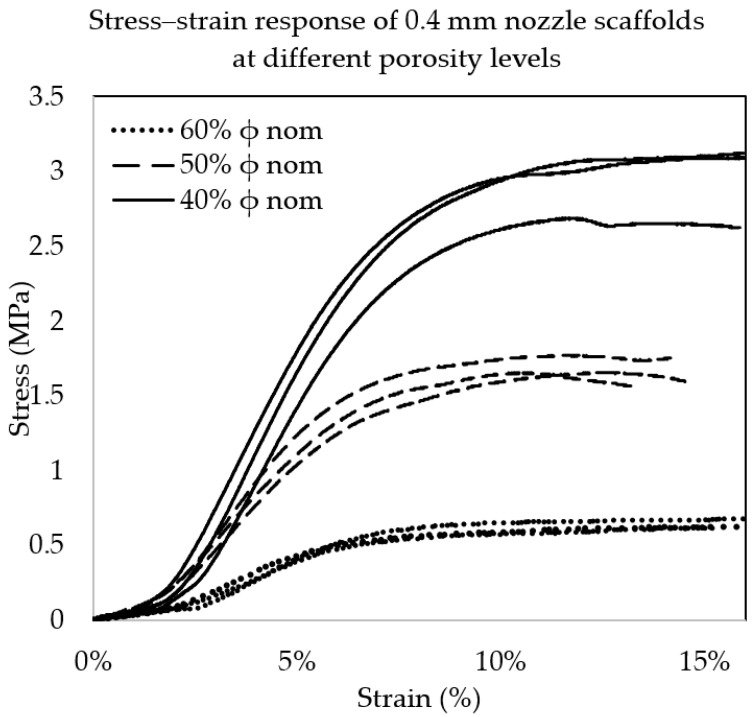
Stress–strain response under uniaxial compression of gyroid scaffolds fabricated with the 0.4 mm nozzle. Three replicates are shown for each nominal porosity level ($\phi_{nom}$ = 40%, 50%, and 60%).

Figure 5 shows the stress–strain curves obtained from uniaxial compression tests of gyroid scaffolds fabricated with the 0.4 mm nozzle. For each nominal porosity level, three experimental replicates are presented. All curves exhibit an initial quasi-linear region followed by a gradual increase in stress with increasing strain up to approximately 15%. As expected, scaffolds with lower porosity sustained higher stress levels under compression than those with higher porosity.

The compressive elastic modulus, E, was determined from the initial linear region (0–2% strain). For the 0.4 mm nozzle, E decreased systematically with increasing porosity. At $\phi_{\mathrm{nom}}=40\%$ (ϕ≈28.0%), E was 49.62±3.22 MPa (n=3). At $\phi_{\mathrm{nom}}=50\%$ (ϕ≈37.33%), E decreased to 32.08±3.16 MPa (n=3). Finally, at $\phi_{\mathrm{nom}}=60\%$ (ϕ≈46.67%), E further decreased to 12.34±0.74 MPa (n=3), indicating a pronounced reduction in effective stiffness with increasing measured porosity. Individual specimen values are reported in Table 8.

#### 3.2.2. 0.3 mm Nozzle

Based on the multi-objective analysis of DoE1, condition M3_11 was selected as the reference configuration for the fabrication of compression specimens using the 0.3 mm nozzle. This parameter combination enabled the production of stable prismatic specimens, free of visible macroscopic defects and compliant with ASTM D695-15 requirements. Accordingly, this condition was directly adopted for DoE2 with the 0.3 mm nozzle.

Under these conditions, nine specimens were fabricated across three nominal porosity levels ($\phi_{\mathrm{nom}}=40\%,\;50\%,\;60\%$; n=3 per level). The measured porosity (ϕ), determined by a densimetric method, was consistently lower than the nominal values. Specifically, measured porosity was approximately 28% for $\phi_{\mathrm{nom}}=40\%$, 37–39% for $\phi_{\mathrm{nom}}=50\%$, and 48–49% for $\phi_{\mathrm{nom}}=60\%$ (Table 9).

The compressive elastic modulus, E, was likewise determined from the initial linear region (0–2% strain) (Table 9). For the 0.3 mm nozzle, E decreased systematically with increasing porosity. At $\phi_{\mathrm{nom}}=40\%$ (ϕ=28.0±1.0%), E was 27.34±3.40 MPa (n=3). At $\phi_{\mathrm{nom}}=50\%$ (ϕ=38.0±1.0%), E decreased to 10.54±1.45 MPa (n=3). Finally, at $\phi_{\mathrm{nom}}=60\%$ (ϕ=48.33±0.58%), E further decreased to 3.79±0.13 MPa (n=3), indicating a pronounced reduction in effective stiffness with increasing measured porosity.

**Figure 6:**
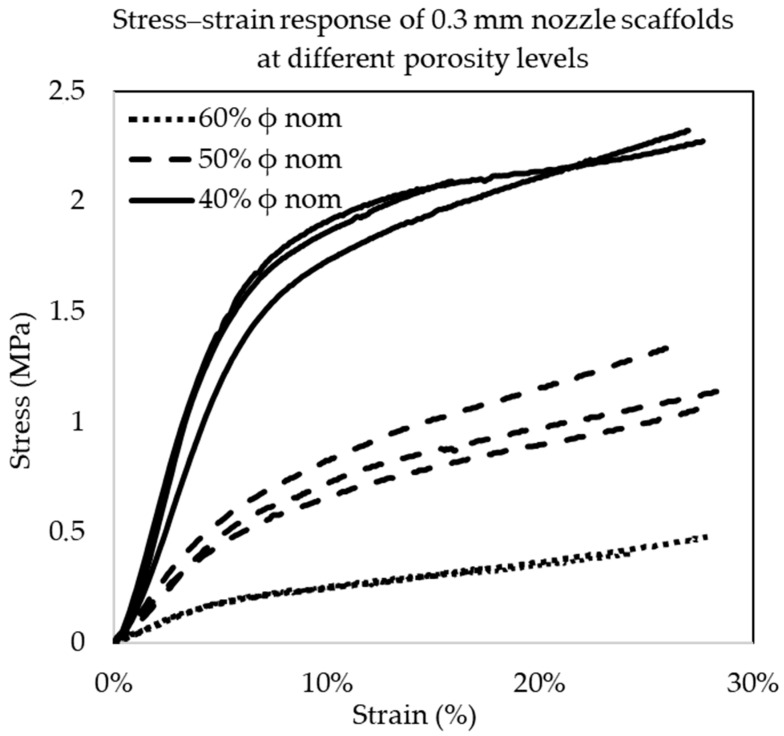
Stress–strain curves obtained from uniaxial compression tests of gyroid scaffolds fabricated with the 0.3 mm nozzle.

Figure 6 shows the stress–strain curves obtained from uniaxial compression tests of gyroid scaffolds fabricated with the 0.3 mm nozzle. As in the 0.4 mm nozzle group, the curves exhibited an initial quasi-linear region followed by a gradual increase in stress with increasing strain. Lower-porosity scaffolds sustained higher stress levels under compression than higher-porosity scaffolds.

### 3.3. Integrated Statistical Analysis

#### 3.3.1. Linear Interaction Model Between Measured Porosity and Nozzle Diameter

The linear interaction model defined in Equation (4) was fitted using measured porosity, ϕ, as a continuous variable and nozzle diameter, $d_{b}$, as a categorical variable coded as $d_{b}=0$ for the 0.3 mm nozzle and $d_{b}=1$ for the 0.4 mm nozzle. The fitted model indicated a decreasing relationship between compressive elastic modulus, E, and measured porosity, ϕ, over the evaluated range (approximately 27–49%). Measured porosity had a significant effect on E ($p=9.77\times 10^{-8}$), and the interaction term $\phi \times d_{b}$ was also significant ($p=2.82\times 10^{-4}$), indicating that the sensitivity of the elastic modulus to changes in measured porosity differed between nozzle diameters. The model explained a large proportion of the observed variability ($R^{2}=0.973$). The estimated coefficients, together with their standard errors and significance levels, are reported in Table 10.

**Figure 7:**
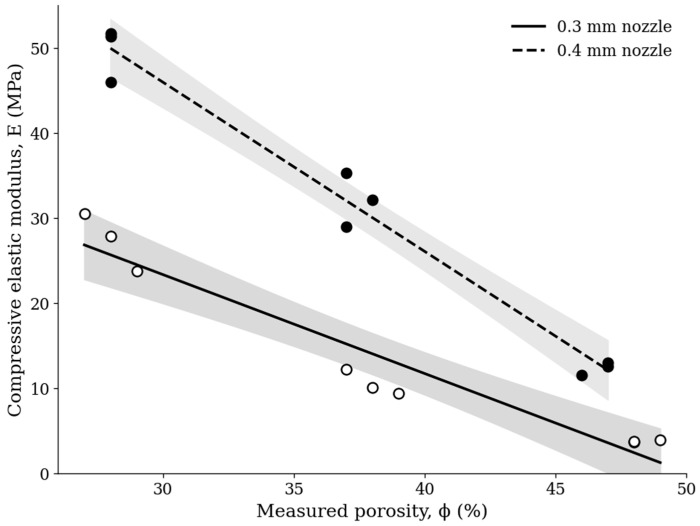
Compressive elastic modulus, E, as a function of measured porosity, ϕ, for FDM-fabricated gyroid PCL scaffolds produced with 0.3 mm and 0.4 mm nozzles (DoE2). The black and white circles correspond to the experimental data reported in Table 8 and Table 9, respectively.

Based on these coefficients, the fitted regression lines for each nozzle diameter were:

- 0.3 mm nozzle: E=58.17−1.162ϕ
- 0.4 mm nozzle: E=105.66−1.991ϕ

where ϕ is expressed as a percentage. Accordingly, the slopes are interpreted in units of MPa/%. Figure 7 shows the experimental values of E as a function of ϕ, together with the fitted linear relationships for each nozzle diameter. The points correspond to individual replicates (n=3 per nominal porosity level and nozzle diameter; total n=18), while the shaded bands represent the 95% confidence interval of the predicted mean.

#### 3.3.2. Effects of Nominal Porosity and Nozzle Diameter on Compressive Elastic Modulus

A two-way analysis of variance (ANOVA) with interaction was performed, considering nozzle diameter ($d_{b}$: 0.3 and 0.4 mm) and nominal porosity ($\phi_{\mathrm{nom}}$: 40%, 50%, and 60%) as categorical factors. The results are summarized in Table 11. The analysis revealed significant effects of both nozzle diameter (F=237.97, $p=2.82\times 10^{-9}$) and nominal porosity (F=242.13, $p=2.00\times 10^{-10}$) on the compressive elastic modulus, E. The interaction term $d_{b}\times \phi_{\mathrm{nom}}$ was also statistically significant (F=15.51, $p=4.71\times 10^{-4}$).

**Figure 8:**
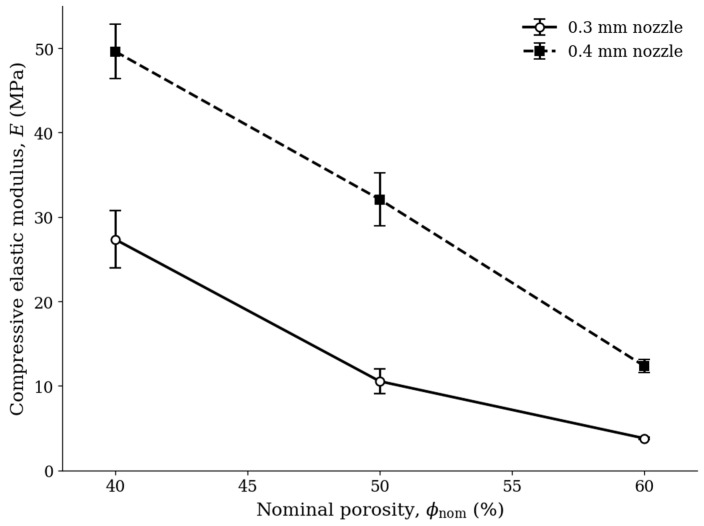
Interaction plot of the compressive elastic modulus, E, as a function of nominal porosity, $\phi_{nom}$, for scaffolds fabricated with 0.3 mm and 0.4 mm nozzles.

From a descriptive standpoint, the compressive elastic modulus, E, increased as nominal porosity decreased (40% > 50% > 60%) for both nozzle diameters. At a given nominal porosity level, specimens fabricated with the 0.4 mm nozzle consistently exhibited higher E values than those produced with the 0.3 mm nozzle (Figure 8). Figure 8 presents the interaction plot of E as a function of $\phi_{\mathrm{nom}}$, where points represent the mean values (n=3) and error bars correspond to the standard deviation.

## 4. Discussion

### 4.1. Pore-Size Deviation and Geometric Fidelity (DoE1)

A systematic mismatch between the nominal pore size and the measured pore size was observed for both nozzle diameters. For the 0.4 mm nozzle, measured pore size ranged from approximately 0.48 to 0.71 mm for a nominal value of 0.8 mm, whereas for the 0.3 mm nozzle it ranged from 0.308 to 0.489 mm for a nominal value of 0.6 mm. In both cases, the measured values were consistently lower than the design values.

This downward bias has been previously reported in extrusion-based PCL scaffolds and is commonly attributed to filament widening relative to the nominal toolpath, which reduces the effective pore opening. Previous studies have described this phenomenon and its direct impact on the final printed architecture [8,22,34]. The present study did not include repeated measurements at different post-fabrication times, so a contribution from post-cooling consolidation cannot be completely excluded. Nevertheless, the consistency of the bias across all evaluated conditions suggests that the main source of mismatch was deposition-related rather than a condition-specific artifact.

#### 4.1.1. The 0.4 mm Nozzle

For the 0.4 mm nozzle, ANOVA identified extrusion temperature as the dominant factor affecting pore size, accounting for 41.5% of the observed variability. This behavior is consistent with previous reports on PCL, in which temperature variations influence material viscosity, extrusion stability, and the effective width of the deposited filament [22]. Lower temperatures may lead to flow instabilities that compromise deposition uniformity and, consequently, pore geometry fidelity.

A relevant finding was the limited transferability of the optimal condition identified in plate specimens to prismatic samples. In DoE1, the M4_18 combination (120 °C, 20 mm/s, 110%) yielded the lowest relative error with respect to the target pore size. However, when applied to the prismatic specimens in DoE2, it resulted in local collapse and geometric distortions that prevented its use in mechanical testing.

This discrepancy suggests that geometric fidelity achieved in thin structures does not necessarily translate into manufacturing stability in bulkier geometries. This interpretation is consistent with previous studies on extrusion-based PCL printing, which have highlighted the sensitivity of the process to thermal stability and deposition control as geometric complexity or specimen volume increases [21,22,27].

#### 4.1.2. The 0.3 mm Nozzle

For the 0.3 mm nozzle, ANOVA identified printing speed as the dominant factor, accounting for 31.6% of the observed variability in pore size. This result is also consistent with previous studies on PCL, in which printing speed has been shown to directly affect filament width and pore size by modifying the balance among material deposition, filament relaxation, and cooling.

For smaller nozzle diameters, this effect becomes more pronounced because the process window is narrower and the deposition is more sensitive to local over-deposition and thermal accumulation. As a result, relatively small changes in printing speed can produce noticeable variations in effective pore opening.

In this context, the present results reinforce the idea that precise control of printing speed is particularly important for achieving geometric fidelity in FDM-fabricated PCL scaffolds produced with finer nozzles [8,21,22].

### 4.2. Compressive Mechanical Response and the Effect of Porosity (DoE2)

In DoE2, the compressive elastic modulus, E, decreased as measured porosity, ϕ, increased for both nozzle diameters. This trend is consistent with the classical behavior of cellular solids, in which macroscopic stiffness decreases as the load-bearing solid fraction and structural connectivity are reduced [35]. The same pattern was clearly observed in both experimental groups, with stiffness progressively decreasing from the lowest to the highest nominal porosity levels.

A relevant finding was that measured porosity was consistently lower than nominal porosity across all evaluated levels and nozzle diameters. This systematic bias is also consistent with previous reports on extrusion-based PCL scaffolds, where variations in effective filament width and final pore opening alter the porosity ultimately achieved after fabrication [8,34,36]. In this context, the present results reinforce the idea that mechanical response should not be interpreted solely based on the nominal CAD-defined geometry, but rather in terms of the architecture actually obtained after fabrication. From a process–structure–property perspective, it is this effective printed architecture, and not the idealized design alone, that governs the initial compressive stiffness of the scaffold.

### 4.3. Statistical Integration of the Process–Structure–Property Relationship

The integrated statistical analysis showed that the relationship between compressive elastic modulus, E, and measured porosity, ϕ, depends on nozzle diameter. In particular, the linear interaction model presented in Section 3.3.1 yielded different slopes for the 0.3 mm and 0.4 mm nozzles and confirmed that the interaction term was statistically significant. Complementarily, the two-way ANOVA presented in Section 3.3.2 also confirmed a significant interaction between nozzle diameter and nominal porosity.

Taken together, these results show that porosity and nozzle diameter interact in their effect on elastic modulus. Accordingly, the same change in porosity does not produce the same stiffness response for both nozzle diameters. Nozzle diameter therefore influences not only the absolute stiffness level, but also the sensitivity of the scaffold to changes in porosity.

From a methodological standpoint, these results further support the use of measured porosity, rather than nominal CAD-defined porosity alone, to interpret mechanical response. In this study, measured porosity was consistently lower than nominal porosity, confirming that the microarchitecture ultimately obtained after fabrication differs from the idealized design. It is this effective printed structure, and particularly its load-bearing solid fraction, which governs scaffold stiffness. Figure 7 and Figure 8 provide a visual summary of the difference between an interpretation based on nominal design and one based on the effective manufactured structure. This interpretation is also consistent with recent efforts to relate deposited geometry to scaffold performance in extrusion-based systems [37].

### 4.4. Exploratory Comparison with Reported Trabecular Bone Stiffness Domains

Because the results showed that scaffold stiffness is governed by the effective printed architecture rather than by nominal design parameters alone, the experimental stiffness ranges obtained in DoE2 can be used for an exploratory mechanical contextualization relative to values reported for trabecular bone. Within the experimental range evaluated, scaffolds fabricated with the 0.4 mm nozzle exhibited elastic modulus values of approximately 11–52 MPa, whereas those produced with the 0.3 mm nozzle ranged from about 3.7 to 30.5 MPa. These intervals partially overlap with stiffness ranges reported for low to moderately stiff trabecular bone, which vary depending on anatomical site and clinical condition [38,39,40].

From this perspective, the present data suggests that stiffness modulation through nozzle diameter and effective printed porosity provides a useful basis for positioning different scaffold configurations within experimentally accessible mechanical ranges. Table 12 presents an exploratory stiffness-based comparison between the DoE2 configurations and reported trabecular bone domains, using the measured compressive elastic modulus as the reference variable. This comparison is intended only as a mechanics-based contextualization of the experimental results and should not be interpreted as a basis for site-specific scaffold selection.

### 4.5. Limitations and Scope of the Study

This study focused on the initial geometric and mechanical characterization of FDM-fabricated gyroid PCL scaffolds under controlled printing conditions and standardized testing geometries. Accordingly, the results describe the quasi-static compressive response and do not account for potentially relevant in-service effects such as anisotropy, fatigue, or cyclic loading. In addition, the internal architecture was characterized through pore-size measurements and densimetric porosity rather than by full three-dimensional imaging techniques, which limits the geometric detail available for interpreting the printed structure.

It should also be noted that the present results correspond to an uncompensated slicing and fabrication workflow. No CAD-based additive manufacturing compensation or G-code correction was applied to anticipate dimensional deviations. Therefore, the absolute magnitude of the pore-size deviations reported here may differ if other printers, slicers, materials, or compensation strategies are used. However, this does not invalidate the observed process–structure–property relationships; rather, it highlights the importance of reporting the effective printed microarchitecture and the slicing conditions when interpreting FDM-fabricated scaffold performance.

The exploratory stiffness-based comparison presented here is also restricted to pure PCL scaffolds and measured geometric descriptors, particularly measured porosity. It therefore does not consider the use of composite materials, bioactive reinforcements, or surface coatings, all of which could alter both the mechanical response and the biological performance of the scaffold and thereby broaden the range of achievable properties.

Finally, although the analysis is discussed in relation to reported trabecular bone stiffness domains, the proposed correspondence should be interpreted as exploratory and mechanics-based only. Biological validation—such as cell adhesion, proliferation, or differentiation—as well as preclinical or clinical assessment of the proposed configurations remain beyond the scope of the present study.

## 5. Conclusions

This study provided an integrated analysis of the effects of FDM processing parameters and nozzle diameter on the printed microarchitecture and compressive response of gyroid PCL scaffolds. A systematic mismatch was observed between the nominal design and the printed structure, with both pore size and measured porosity consistently lower than their intended values. These findings highlight the need to rely on measured geometric descriptors, rather than nominal CAD-defined parameters alone, when interpreting the mechanical performance of FDM-fabricated scaffolds.

DoE1 showed that the dominant process parameter associated with pore-size variability differed between nozzle groups: extrusion temperature showed the largest contribution for the 0.4 mm nozzle, whereas printing speed showed the largest contribution for the 0.3 mm nozzle. In DoE2, the compressive elastic modulus, E, decreased with increasing measured porosity, ϕ, and the integrated statistical analysis confirmed that the E–ϕ relationship was nozzle-dependent. Taken together, these results show that the mechanical response of these scaffolds cannot be explained solely on the basis of nominal design parameters but instead should be interpreted within a process–structure–property framework grounded in the effective printed microarchitecture.

Based on the experimental stiffness ranges obtained and the observed relationship between E and measured porosity, an exploratory stiffness-based comparison was established relative to trabecular bone domains reported in the literature. This comparison should be interpreted only as a mechanical contextualization of the experimental scaffold ranges, rather than as evidence for site-specific scaffold selection or application.

Future work should incorporate anisotropy and fatigue or cyclic loading effects, as well as biological validation of the proposed configurations, for example in terms of cell adhesion and proliferation. It would also be valuable to extend this framework to composite scaffolds, such as PCL/β-TCP, and to validate the printed microarchitecture through three-dimensional imaging so as to assess how material modification and geometric fidelity jointly alter the process–structure–property relationship and the resulting stiffness-based design space.

## Figures and Tables

**Table 1:** Experimental levels of the FDM process parameters evaluated for each nozzle diameter.

Factor	Unit	0.4 mm Nozzle	0.3 mm Nozzle
Extrusion temperature (T)	°C	80, 100, 120	90, 100, 110
Printing speed (V)	mm/s	5, 10, 20	5, 7.5, 10
Material flow rate (F)	% of nominal Cura extrusion	100, 105, 110	95, 100, 105

**Table 2:** Fixed slicing parameters used in Ultimaker Cura for the fabrication of CAD-defined gyroid PCL scaffolds.

Slicing Parameter	0.4 mm Nozzle	0.3 mm Nozzle
Layer height	0.20 mm	0.15 mm
Infill density	100%	100%
Cura infill pattern	Not used to define scaffold architecture	Not used to define scaffold architecture
Build plate temperature	0 °C	0 °C
Cooling fan	Disabled	Disabled
Retraction	Disabled	Disabled
Infill line overlap	Not intentionally applied/default Cura setting	Not intentionally applied/default Cura setting
Horizontal expansion	0 mm/not intentionally applied	0 mm/not intentionally applied
Hole horizontal expansion	0 mm/not intentionally applied	0 mm/not intentionally applied
Travel speed	Equal to printing speed	Equal to printing speed
Ambient temperature	20 °C	20 °C

**Table 3:** Taguchi L18 experimental combinations for the 0.4 mm and 0.3 mm nozzles.

0.4 mm Nozzle				0.3 mm Nozzle			
Condition	T (°C)	V (mm/s)	F (%)	Condition	T (°C)	V (mm/s)	F (%)
M4_1	80	5	110	M3_1	90	5	95
M4_2	80	10	100	M3_2	90	5	105
M4_3	80	20	105	M3_3	90	7.5	95
M4_4	80	5	105	M3_4	90	7.5	100
M4_5	80	10	110	M3_5	90	10	95
M4_6	80	20	100	M3_6	90	10	105
M4_7	100	5	100	M3_7	100	5	100
M4_8	100	10	105	M3_8	100	5	105
M4_9	100	20	110	M3_9	100	7.5	95
M4_10	100	5	110	M3_10	100	7.5	100
M4_11	100	10	100	M3_11	100	10	100
M4_12	100	20	105	M3_12	100	10	105
M4_13	120	5	105	M3_13	110	5	95
M4_14	120	10	110	M3_14	110	5	105
M4_15	120	20	100	M3_15	110	7.5	100
M4_16	120	5	100	M3_16	110	7.5	105
M4_17	120	10	105	M3_17	110	10	100
M4_18	120	20	110	M3_18	110	10	105

**Table 4:** Printing parameters and summary statistics of pore size for the 18 conditions fabricated with the 0.4 mm nozzle.

Condition	T (°C)	V (mm/s)	F (%)	${\bar{P}}_{i}$ (mm)	$s_{i}$ (mm)	$e_{i}$ (%)
M4_1	80	5	110	0.550	0.035	31
M4_2	80	10	100	0.573	0.032	28
M4_3	80	20	105	0.535	0.045	33
M4_4	80	5	105	0.588	0.035	26
M4_5	80	10	110	0.534	0.029	33
M4_6	80	20	100	0.481	0.057	40
M4_7	100	5	100	0.566	0.047	29
M4_8	100	10	105	0.614	0.030	23
M4_9	100	20	110	0.662	0.032	17
M4_10	100	5	110	0.508	0.084	37
M4_11	100	10	100	0.643	0.061	20
M4_12	100	20	105	0.616	0.060	23
M4_13	120	5	105	0.570	0.042	29
M4_14	120	10	110	0.650	0.049	19
M4_15	120	20	100	0.649	0.027	19
M4_16	120	5	100	0.633	0.072	21
M4_17	120	10	105	0.659	0.016	18
M4_18	120	20	110	0.709	0.019	11

Note: ${\bar{P}}_{i}$ is the condition-level mean pore size, $s_{i}$ is the inter-specimen standard deviation, and $e_{i}$ is the relative error between the measured mean pore size and the nominal target pore size. The subscript i identifies each experimental condition.

**Table 5:** ANOVA results for specimen-level average pore size in scaffolds fabricated with the 0.4 mm nozzle (n=54).

Source	Sum of Squares (SS)	df	Mean Square (MS)	F-Value	*p*-Value	Contribution (%)
Temperature (T)	0.093687	2	0.0582	19.9	$5.439\times 10^{-7}$	41.5
Printing speed (V)	0.020450	2	0.0127	4.34	$1.84\times 10^{-2}$	9.1
Flow rate (F)	0.001175	2	0.0007	0.25	$7.825\times 10^{-1}$	0.5
Residual	0.110637	47	0.0029	—	—	48.9

**Table 6:** Printing parameters and summary statistics of pore size for the 18 conditions fabricated with the 0.3 mm nozzle.

Condition	T (°C)	V (mm/s)	F (%)	${\bar{P}}_{i}$ (mm)	$s_{i}$ (mm)	$e_{i}$ (%)
M3_1	90	5.0	95	0.348	0.032	42
M3_2	90	5.0	105	0.350	0.041	42
M3_3	90	7.5	95	0.308	0.053	49
M3_4	90	7.5	100	0.337	0.030	44
M3_5	90	10.0	95	0.356	0.062	41
M3_6	90	10.0	105	0.349	0.037	42
M3_7	100	5.0	100	0.397	0.038	34
M3_8	100	5.0	105	0.337	0.035	44
M3_9	100	7.5	95	0.358	0.020	40
M3_10	100	7.5	100	0.351	0.026	42
M3_11	100	10.0	100	0.489	0.046	19
M3_12	100	10.0	105	0.406	0.064	32
M3_13	110	5.0	95	0.315	0.032	48
M3_14	110	5.0	105	0.334	0.050	44
M3_15	110	7.5	100	0.352	0.043	41
M3_16	110	7.5	105	0.347	0.016	42
M3_17	110	10.0	100	0.415	0.045	31
M3_18	110	10.0	105	0.410	0.029	32

Note: ${\bar{P}}_{i}$ is the condition-level mean pore size, $s_{i}$ is the inter-specimen standard deviation, and $e_{i}$ is the relative error between the measured mean pore size and the nominal target pore size. The subscript i identifies each experimental condition.

**Table 7:** ANOVA results for specimen-level average pore size in scaffolds fabricated with the 0.3 mm nozzle (n=54).

Source	Sum of Squares (SS)	df	Mean Square (MS)	F-Value	*p*-Value	Contribution (%)
Temperature (T)	0.01090	2	0.00545	3.72	3.16 × 10^−2^	8.2
Printing speed (V)	0.04182	2	0.02091	14.28	1.43 × 10^−5^	31.6
Flow rate (F)	0.01092	2	0.00546	3.73	3.14 × 10^−2^	8.2
Residual	0.06884	47	0.00147	—	—	52.0

**Table 8:** Nominal porosity, measured porosity, and compressive elastic modulus (E) of specimens fabricated with the 0.4 mm nozzle.

Test	$\phi_{nom}$ (%)	ϕ (%)	E (MPa)
1	60	47	12.98
2	60	46	11.53
3	60	47	12.52
4	50	37	28.93
5	50	38	32.06
6	50	37	35.24
7	40	28	45.91
8	40	28	51.67
9	40	28	51.29

**Table 9:** Nominal porosity, measured porosity, and compressive elastic modulus (E) of specimens fabricated with the 0.3 mm nozzle.

Test	$\phi_{nom}$ (%)	*ϕ* (%)	E (MPa)
1	60	48	3.68
2	60	49	3.94
3	60	48	3.75
4	50	38	10.06
5	50	37	12.17
6	50	39	9.40
7	40	28	27.80
8	40	27	30.49
9	40	29	23.73

**Table 10:** Estimated coefficients of the linear interaction model for the relationship between E and measured porosity.

Term	Symbol	Coefficient	Standard Error	t	*p*-Value
Intercept (0.3 mm nozzle)	$\beta_{0}$	58.1677	4.5485	12.788	4.12 × 10^−9^
Measured porosity (0.3 mm nozzle)	$\beta_{1}$	−1.1618	0.1166	−9.964	9.77 × 10^−8^
Nozzle diameter (0.4 mm vs. 0.3 mm)	$\beta_{2}$	47.4936	6.6505	7.141	5.00 × 10^−6^
$\mathrm{Interaction}\;\phi \times d_{b}$	$\beta_{3}$	−0.8288	0.1726	−4.800	2.82 × 10^−4^

Note: ϕ is expressed in %. Nozzle diameter was coded as $d_{b}=0$ for the 0.3 mm nozzle and $d_{b}=1$ for the 0.4 mm nozzle. Therefore, the intercept for the 0.4 mm nozzle is $\beta_{0}+\beta_{2}$, and the slope for the 0.4 mm nozzle is $\beta_{1}+\beta_{3}$.

**Table 11:** Results of the two-way ANOVA with interaction for the compressive elastic modulus, *E*.

Source	df	SS	MS	F	*p*-Value
Nozzle diameter ($d_{b}$)	1	1371.31	1371.31	237.97	2.82 × 10^−9^
Nominal porosity ($\phi_{nom}$)	2	2790.65	1395.32	242.13	2.00 × 10^−10^
($d_{b}$ × $\phi_{nom}$)	2	178.78	89.39	15.51	4.71 × 10^−4^
Error	12	69.15	5.76	—	—
Total	17	4409.89	—	—	—

**Table 12:** Exploratory stiffness-based comparison between DoE2 scaffold configurations and reported trabecular bone domains.

Site/Tissue	Clinical Context	DoE2 Configuration	Measured E in This Study (MPa)	Exploratory Stiffness-Based Interpretation	References
Vertebral body	Osteoporosis	$d_{b}$ = 0.4 mm $\phi_{nom}=40\%$	49.62 ± 3.22	Upper experimental stiffness range; partial overlap with compromised vertebral trabecular bone values	[38,40,41]
Iliac crest	Older adult	$d_{b}$ = 0.4 mm $\phi_{nom}=50\%$	32.08 ± 3.16	Intermediate experimental stiffness range; partial overlap with reported iliac trabecular values	[39,42]
Iliac crest	Older adult	$d_{b}$ = 0.3 mm $\phi_{nom}=40\%$	27.34 ± 3.40	Intermediate lower-stiffness alternative; partial overlap with reported iliac trabecular values	[39,43]
Calcaneus	No osteoporosis (reference)	$d_{b}$ = 0.4 mm $\phi_{nom}=50\%/d_{b}$ = 0.3 mm $\phi_{nom}=40\%$	32.08 ± 3.16/27.34 ± 3.40	Intermediate experimental range; partial overlap with reported calcaneal trabecular values	[44,45,46,47]
Calcaneus	Osteoporosis/low bone quality	$d_{b}$ = 0.4 mm $\phi_{nom}=60\%/d_{b}$ = 0.3 mm $\phi_{nom}=50\%$	12.34 ± 0.74/10.54 ± 1.45	Lower experimental stiffness range; preliminary reference for softer trabecular domains	[45,46,47]
Maxillofacial (alveolar bone)	Anterior maxilla/posterior mandible	$d_{b}$ = 0.4 mm $\phi_{nom}=40\%$	49.62 ± 3.22	Highest stiffness among the pure PCL configurations evaluated; approaches the upper range of some reported alveolar bone values	[48,49]
Maxillofacial (alveolar bone)	Posterior maxilla	$d_{b}$ = 0.4 mm $\phi_{nom}=50\%$ /$d_{b}$ = 0.3 mm $\phi_{nom}=40\%$	32.08 ± 3.16/27.34 ± 3.40	Intermediate experimental range; partial overlap with less stiff alveolar trabecular values	[48,49,50]
Maxillofacial (high-load)	Segmental defects	Not evaluated with pure PCL	—	Not resolved within the present experimental range; higher-load cases may require stiffer materials or architectures	[19,51]

Note: The comparisons presented in this table are based exclusively on the initial compressive elastic modulus measured in DoE2. Because reported trabecular bone stiffness values vary with anatomical site, age, bone quality, density, and test methodology, these comparisons should be interpreted only as an exploratory mechanical contextualization of the experimental scaffold ranges, not as a basis for site-specific scaffold selection or clinical indication.

## Data Availability

The original contributions presented in this study are included in the article/Supplementary Material. Further inquiries can be directed to the corresponding author.

## Supplementary Materials

The following supporting information can be downloaded at: https://www.mdpi.com/article/10.3390/jfb17060289/s1, Table S1: Raw pore size measurements for scaffolds fabricated with the 0.4 mm nozzle (DoE1); Table S2: Summary statistics of pore size per condition for scaffolds fabricated with the 0.4 mm nozzle (DoE1); Table S3: Raw pore size measurements for scaffolds fabricated with the 0.3 mm nozzle (DoE1); Table S4: Summary statistics of pore size per condition for scaffolds fabricated with the 0.3 mm nozzle (DoE1).
