# Supplementary material for: Influence of Printing Parameters and Nozzle Diameter on the Effective Microarchitecture and Compressive Modulus of Gyroid PCL Scaffolds
Source: J Funct Biomater. 2026 Jun 9;17(6):289. doi: 10.3390/jfb17060289 (PMC13302465; doi:10.3390/jfb17060289)
Supplement: Supplementary file 1 [file jfb-17-00289-s001.zip › jfb-4310331-supplementary.pdf]

Supplementary Material

Table S1. Raw pore size measurements for scaffolds fabricated with the 0.4 mm nozzle (DoE1).

**Scaffold**	**Specimen 1**			**Specimen 2**			**Specimen 3**		
	M1	M2	M3	M1	M2	M3	M1	M2	M3
M4_1	0.487	0.527	0.567	0.544	0.555	0.596	0.561	0.521	0.595
M4_2	0.573	0.586	0.505	0.623	0.611	0.548	0.555	0.573	0.579
M4_3	0.468	0.532	0.589	0.532	0.524	0.581	0.573	0.484	0.529
M4_4	0.624	0.596	0.667	0.598	0.538	0.614	0.523	0.580	0.554
M4_5	0.567	0.580	0.525	0.505	0.518	0.525	0.511	0.580	0.491
M4_6	0.452	0.339	0.532	0.573	0.452	0.500	0.478	0.502	0.500
M4_7	0.556	0.512	0.568	0.543	0.556	0.661	0.514	0.599	0.587
M4_8	0.651	0.667	0.616	0.635	0.561	0.598	0.623	0.572	0.604
M4_9	0.694	0.673	0.633	0.602	0.633	0.684	0.653	0.694	0.696
M4_10	0.555	0.574	0.406	0.515	0.347	0.509	0.627	0.502	0.535
M4_11	0.599	0.651	0.661	0.576	0.657	0.740	0.587	0.710	0.604
M4_12	0.649	0.536	0.712	0.642	0.620	0.614	0.508	0.659	0.608
M4_13	0.550	0.462	0.497	0.544	0.636	0.623	0.609	0.570	0.636
M4_14	0.611	0.720	0.652	0.658	0.612	0.702	0.601	0.686	0.607
M4_15	0.696	0.630	0.671	0.622	0.705	0.647	0.619	0.630	0.622
M4_16	0.601	0.754	0.722	0.608	0.544	0.620	0.538	0.722	0.589
M4_17	0.680	0.654	0.647	0.660	0.640	0.673	0.660	0.647	0.673
M4_18	0.709	0.701	0.714	0.789	0.722	0.715	0.689	0.676	0.667

Table S2. Summary statistics of pore size per condition for scaffolds fabricated with the 0.4 mm nozzle (DoE1).

**Scaffold**	$\overline{P}_{i1}$	s_{i1}	$\overline{P}_{i2}$	s_{i2}	$\overline{P}_{i3}$	s_{i3}	$\overline{P}_i$ (mm)	s_i (mm)	e_i (%)
M4_1	0.527	0.040	0.565	0.027	0.559	0.037	0.550	0.035	31
M4_2	0.555	0.044	0.594	0.040	0.569	0.012	0.573	0.032	28
M4_3	0.530	0.061	0.546	0.031	0.529	0.045	0.535	0.045	33
M4_4	0.629	0.036	0.583	0.040	0.552	0.029	0.588	0.035	26
M4_5	0.557	0.029	0.516	0.010	0.527	0.047	0.534	0.029	33
M4_6	0.441	0.097	0.508	0.061	0.493	0.013	0.481	0.057	40
M4_7	0.545	0.029	0.587	0.065	0.567	0.046	0.566	0.047	29
M4_8	0.645	0.026	0.598	0.037	0.600	0.026	0.614	0.030	23
M4_9	0.667	0.031	0.640	0.041	0.681	0.024	0.662	0.032	17
M4_10	0.512	0.092	0.457	0.095	0.555	0.065	0.508	0.084	37
M4_11	0.637	0.033	0.658	0.082	0.634	0.067	0.643	0.061	20
M4_12	0.632	0.089	0.625	0.015	0.592	0.077	0.616	0.060	23
M4_13	0.503	0.044	0.601	0.050	0.605	0.033	0.570	0.042	29
M4_14	0.661	0.055	0.657	0.045	0.631	0.047	0.650	0.049	19
M4_15	0.666	0.033	0.658	0.043	0.624	0.006	0.649	0.027	19
M4_16	0.692	0.081	0.591	0.041	0.616	0.095	0.633	0.072	21
M4_17	0.660	0.017	0.658	0.017	0.660	0.013	0.659	0.016	18
M4_18	0.708	0.007	0.742	0.041	0.677	0.011	0.709	0.019	11

Table S3. Raw pore size measurements for scaffolds fabricated with the 0.3 mm nozzle (DoE1).

Scaffold	Specimen 1			Specimen 2			Specimen 3		
	M1	M2	M3	M1	M2	M3	M1	M2	M3
M3_1	0,278	0,373	0,370	0,368	0,378	0,341	0,332	0,325	0,370
M3_2	0,307	0,405	0,379	0,333	0,281	0,353	0,399	0,327	0,366
M3_3	0,368	0,306	0,245	0,336	0,266	0,353	0,249	0,298	0,352
M3_4	0,347	0,331	0,385	0,287	0,331	0,341	0,374	0,325	0,309
M3_5	0,337	0,419	0,375	0,424	0,324	0,262	0,291	0,357	0,419
M3_6	0,315	0,411	0,315	0,397	0,343	0,342	0,315	0,336	0,363
M3_7	0,344	0,438	0,370	0,431	0,485	0,404	0,343	0,364	0,391
M3_8	0,373	0,336	0,365	0,250	0,312	0,291	0,307	0,393	0,410
M3_9	0,265	0,278	0,290	0,456	0,409	0,444	0,377	0,369	0,334
M3_10	0,330	0,363	0,371	0,380	0,310	0,348	0,331	0,373	0,353
M3_11	0,462	0,436	0,524	0,462	0,433	0,529	0,567	0,490	0,495
M3_12	0,441	0,386	0,525	0,446	0,337	0,343	0,455	0,337	0,383
M3_13	0,243	0,297	0,257	0,368	0,289	0,332	0,357	0,374	0,317
M3_14	0,337	0,372	0,372	0,413	0,419	0,279	0,213	0,306	0,298
M3_15	0,457	0,350	0,329	0,291	0,286	0,338	0,336	0,386	0,393
M3_16	0,302	0,356	0,307	0,413	0,409	0,391	0,316	0,307	0,321
M3_17	0,351	0,466	0,412	0,466	0,453	0,385	0,374	0,437	0,387
M3_18	0,473	0,401	0,423	0,413	0,373	0,420	0,373	0,423	0,394

Table S4. Summary statistics of pore size per condition for scaffolds fabricated with the 0.3 mm nozzle (DoE1).

Scaffold	$\overline{P}_{i1}$	s_{i1}	$\overline{P}_{i2}$	s_{i2}	$\overline{P}_{i3}$	s_{i3}	$\overline{P}_i$ (mm)	s_i (mm)	e_i (%)
M3_1	0,340	0,054	0,362	0,019	0,342	0,024	0,348	0,032	42%
M3_2	0,364	0,051	0,322	0,037	0,364	0,036	0,350	0,041	42%
M3_3	0,306	0,062	0,318	0,046	0,300	0,052	0,308	0,053	49%
M3_4	0,354	0,028	0,320	0,029	0,336	0,034	0,337	0,030	44%
M3_5	0,377	0,041	0,337	0,082	0,356	0,064	0,356	0,062	41%
M3_6	0,347	0,055	0,361	0,031	0,338	0,024	0,349	0,037	42%
M3_7	0,384	0,049	0,440	0,041	0,366	0,024	0,397	0,038	34%
M3_8	0,358	0,019	0,284	0,032	0,370	0,055	0,337	0,035	44%
M3_9	0,278	0,013	0,436	0,024	0,360	0,023	0,358	0,020	40%
M3_10	0,355	0,022	0,346	0,035	0,352	0,021	0,351	0,026	42%
M3_11	0,474	0,045	0,475	0,049	0,517	0,043	0,489	0,046	19%
M3_12	0,451	0,070	0,375	0,061	0,392	0,059	0,406	0,064	32%
M3_13	0,266	0,028	0,330	0,040	0,349	0,029	0,315	0,032	48%
M3_14	0,360	0,020	0,370	0,079	0,272	0,052	0,334	0,050	44%
M3_15	0,379	0,069	0,305	0,029	0,372	0,031	0,352	0,043	41%
M3_16	0,322	0,030	0,404	0,012	0,315	0,007	0,347	0,016	42%
M3_17	0,410	0,058	0,435	0,044	0,399	0,033	0,415	0,045	31%
M3_18	0,432	0,037	0,402	0,025	0,397	0,025	0,410	0,029	32%
